# Three Siblings With Familial Isolated Hypoparathyroidism: A Diagnostic Journey From *CASR* to Novel *GCM2* Variant

**DOI:** 10.1210/jcemcr/luae185

**Published:** 2024-10-22

**Authors:** Apisadaporn Thambundit, Julian A Martinez-Agosto, Jessica Kianmahd Shamshoni, Karen K Winer, Steven D Mittelman

**Affiliations:** Division of Pediatric Endocrinology, UCLA Children's Discovery and Innovation Institute, David Geffen School of Medicine, University of California Los Angeles, Los Angeles, CA 90095, USA; Department of Human Genetics, UCLA, Los Angeles, CA 90095, USA; Department of Human Genetics, UCLA, Los Angeles, CA 90095, USA; Eunice Kennedy Shriver National Institutes of Child Health and Human Development, National Institutes of Health, Bethesda, MD 20892, USA; Division of Pediatric Endocrinology, UCLA Children's Discovery and Innovation Institute, David Geffen School of Medicine, University of California Los Angeles, Los Angeles, CA 90095, USA

**Keywords:** familial isolated hypoparathyroidism, parathyroid hormone, hypocalcemia, GCM2

## Abstract

We report a patient who initially presented at 4 days old with hypocalcemia, hypoparathyroidism, and elevated phosphorous level. Treatment was initiated with calcitriol, calcium carbonate (CaCO_3_), vitamin D, and low phosphorous formula. Family history was positive for an activating calcium sensing receptor (*CASR*) variant (R990G) identified previously in 2 older siblings who were treated with CaCO_3_ and calcitriol. However, genetic studies were negative for the *CASR* variant in our patient. She maintained a large calcium requirement and was admitted for multiple episodes of hypocalcemia. Further investigation revealed that the *CASR* variant identified in the older siblings was now considered a benign, nondisease-causing variant. Whole exome sequencing on our proband revealed a homozygous pathogenic variant in the *GCM2* gene (Gln392*) consistent with a molecular diagnosis of familial isolated hypoparathyroidism. Genetic studies revealed the 2 older siblings harbor the same genetic changes and parents are heterozygous carriers for this allele. Due to persistent hypocalcemia, we initiated teriparatide. She weaned off calcitriol and achieved normocalcemia on teriparatide, CaCO_3_, and vitamin D. Siblings transitioned to the same treatment without complications. These findings demonstrate the importance of adequate diagnostic genetic testing and the role of variant reanalysis over time in promoting accurate diagnoses.

## Introduction

Hypoparathyroidism (HP) is rare in pediatrics and can occur as an isolated endocrinopathy (familial isolated hypoparathyroidism [FIH]), or as a component of a complex disorder. Absent or insufficient parathyroid hormone (PTH) leads to hypocalcemia, hyperphosphatemia, and an inability to convert vitamin D to its active form, 1,25(OH)_2_ vitamin D. Patients are generally treated with calcium (Ca) and calcitriol to maintain Ca levels. However, FIH is often difficult to manage with widely fluctuating Ca levels despite high doses of medications, placing patients at risk for complications associated with severe hypocalcemia including seizures, arrhythmias, and laryngospasms [1]; chronic treatment with Ca and calcitriol can lead to nephrocalcinosis and impaired renal function [2].

FIH can occur sporadically or be caused by defects in genes that play an important role in embryologic development of the parathyroid gland, such as the *PTH* gene or glial cells missing homolog 2 (*GCM2)* gene. FIH can also be caused by an activating pathogenic variant in the calcium sensing receptor (*CASR*); inappropriate activation of this sensor signals Ca sufficiency even during hypocalcemia, thereby suppressing PTH secretion. In addition, the abnormal CASR contributes to renal Ca wasting [3].

We report herein a set of 3 siblings who were initially diagnosed with a *CASR* pathogenic variant but were found to be homozygous for a novel pathogenic variant in the *GCM2* gene causing FIH. Additionally, we report the successful management of these siblings with teriparatide.

## Case Presentation

The patient presented at 4 days old in the pediatric endocrinology clinic to screen for possible hypocalcemia. She was born at an outside hospital at 38 weeks gestational age with a birthweight of 3.4 kilograms to healthy, nonconsanguineous Hispanic Caucasian parents. There was no neonatal stress, and physical exam did not note dysmorphic features, jitteriness, or irritability. She was discharged home from the newborn nursery on day 2 of life, and the parents denied any twitching or seizure-like movements. She was feeding well with breast milk and Similac Advance formula 2 to 3 ounces every 2 to 3 hours. She had an adequate amount of urine and stool output. Her siblings, aged 11 and 9 at the time, had been diagnosed with an activating *CASR* pathogenic variant (R990G), so the parents wanted to ensure the patient did not have the same condition. The siblings were managed with calcitriol (0.018-0.03 µg/kg/d) and calcium carbonate (CaCO_3_) supplementation (38-100 mg elemental Ca/kg/day).

## Diagnostic Assessment

The physical exam was benign with appropriate growth parameters and no signs of severe hypocalcemia. Labs were consistent with HP: total Ca 6.8 mg/dL (1.7 mmol/L) [reference range (RR): 8.6-10.3 mg/dL; 2.15-2.57 mmol/L], PTH 5 pg/mL (0.53 pmol/L) (RR: 11-51 pg/mL; 1.17-5.41 pmol/L), magnesium 1.2 mEq/L (0.6 mmol/L) (RR: 1.4-1.9 mEq/L; 0.7-0.95 mmol/L), and phosphorous 7.9 mg/dL (2.6 mmol/L) (RR: 3.9-6.9 mg/dL; 1.3-2.2 mmol/L). Genetic studies were sent for *CASR* sequencing.

## Treatment

The patient was presumed to have HP secondary to hypocalcemia and hyperphosphatemia with low PTH and the same *CASR* R990G variant as her siblings. She was started on calcitriol 0.03 µg/kg daily, CaCO_3_ 80 mg elemental Ca/kg/day, and vitamin D 400 international units daily and switched to low-phosphorous formula, Similac 60/40. A urine calcium and creatinine ratio was ordered, but collection was unsuccessful. A renal ultrasound was not obtained by the physician caring for the patient at the time.

## Outcome and Follow-up

At 2 months of age, genetic testing did not detect abnormal variant in the *CASR* gene. Ca levels remained 10 mg/dL or higher, so she transitioned back to standard formula, calcitriol and vitamin D were discontinued, and CaCO_3_ was weaned down to 34 mg elemental Ca/kg/day. She maintained normal Ca levels from age 3 to 7 months on this low CaCO_3_ dose before Ca levels began to decline despite increasing Ca supplementation. At 11 months of age, she presented with generalized tonic clonic seizures associated with high-grade fever and severe hypocalcemia. Total serum Ca was 5.1 mg/dL (1.27 mmol/L) (RR: 8.6-10.3 mg/dL; 2.15-2.57 mmol/L) and phosphorous was 11.2 mg/dL (3.6 mmol/L) (RR: 4.6-7.9 mg/dL; 1.5-2.5 mmol/L). She was treated with Ca gluconate intravenously until normalization of calcium levels. Calcitriol was restarted and CaCO_3_ was increased.

The patient continued to have hypocalcemia requiring high doses of supplementation up to 96 mg elemental Ca/kg/day. The urine calcium to creatinine ratio had been normal for her age, and renal ultrasound was unremarkable. At 15 months old, she presented with left foot tremors and vomiting due to acute gastroenteritis. Total calcium was low at 5.7 mg/dL (1.42 mmol/L) (RR: 8.6-10.3 mg/dL; 2.15-2.57 mmol/L), and the urine calcium to creatinine ratio was elevated at 0.55 mg/mg (RR: <0.2 mg/mg) so hydrochlorothiazide was added at 10 mg/kg/day, which reduced urine calcium to creatinine ratio to 0.12 mg/mg (RR: <0.2 mg/mg) in 2 days. Given the adverse effect of high-dose CaCO_3_, elevated urine calcium to creatine ratio, and inability to maintain stable serum calcium levels, teriparatide was started at 0.7 µg/kg/day subcutaneously divided twice daily. Within 15 hours of administration, her total calcium level increased from 6.9 mg/dL (1.72 mmol) to 8.9 mg/dL (2.22 mmol) (RR: 8.6-10.3 mg/dL; 2.15-2.57 mmol/L). Within 2 days, hydrochlorothiazide was weaned off and calcitriol and CaCO_3_ doses decreased. The patient was discharged on teriparatide 0.8 µg/kg/day, calcitriol 0.02 µg/kg/day, and CaCO_3_ 50 mg elemental Ca/kg/day.

Given the patient's clear HP yet negative detection for a genetic variant in the *CASR* gene, additional genetic investigation was pursued. Whole exome sequencing revealed a homozygous variant in the *GCM2* gene (Gln392*) consistent with a molecular diagnosis of FIH. Further evaluation revealed that the 2 older siblings harbor the same homozygous variant in *GCM2* and both parents are heterozygous carriers for this allele. Additionally, we confirmed that our patient does have the same *CASR* R990G variant as her siblings. However, the analysis was reported as negative because this genetic variant had been reclassified as nonpathogenic.

The patient and both siblings have since been managed with teriparatide, CaCO_3_, and vitamin D. Although there are still fluctuations in total serum and urine Ca levels, the total CaCO_3_ requirement is lower, and all patients are off calcitriol. The patient is now 8 years old with appropriate growth and development. Since the initiation of teriparatide, she only had 1 further hospitalization for severe hypocalcemia associated with high fever at age 30 months. Of note, the patient presented with generalized tonic clonic seizures at 6 years old not associated with hypocalcemia. Her seizures were noted to be different than those associated with hypocalcemia and lasted 3 minutes followed by a post-ictal period. She did not require antiepileptic drugs as she returned to baseline upon arrival to the emergency department. Serum Ca was 8.2 mg/dL (2.0 mmol) (RR: 8.6-10.3 mg/dL; 2.15-2.57 mmol/L), complete blood count, and comprehensive metabolic panel were normal. Magnetic resonance imaging brain with and without contrast was unremarkable. She was discharged with neurology follow-up and rescue antiepileptic drugs. A 2-hour outpatient electroencephalogram was normal 3 months after presentation, and she has had no further seizures to date. Given the normal Ca level at the time of seizure, a negative family history of seizures, and normal electroencephalograms, the etiology remains unclear.

## Discussion

Isolated HP is a rare disease that presents early in life as asymptomatic disease or life-threatening hypocalcemia. A variety of genes have been associated with isolated HP [4-6]. For example, loss of function variants in the glial cell missing homolog 2 gene (*GCM2*, previously *GCMB*) or SRY-box transcription factor 3 (*SOX3*) impair parathyroid gland development, while pathogenic variants in the *CASR* and *PTH* genes impair PTH secretion. *CASR* activating variants can also impair renal Ca reabsorption, further complicating repletion efforts [5, 6].

The *GCM2* gene is located on chromosome 6p24.2 and has 5 exons including a DNA-binding domain, 2 putative transactivation domains, a putative inhibitory domain, and 1 domain of unknown function. It is expressed mainly in the developing and mature parathyroid glands and encodes a transcription factor that serves as a master regulator of parathyroid gland development. Genetically engineered mice lacking *GCM2* developed HP due to parathyroid gland aplasia, proving its role in parathyroid organogenesis and homeostasis [7]. In humans, pathogenic variants have been discovered in exons 2, 3, and 5 with homozygous loss causing HP. Variants in these domains seem to interfere with DNA binding or transactivational activity [5, 6, 8, 9].

Our patient and her siblings were found to have a homozygous c. 1174C > T (NM_004752.3) variant inherited from each parent that introduces a premature stop codon (Gln392*) in *GCM2*, resulting in a protein lacking its DNA binding domain. This would be predicted to impair its ability to control the expression of genes required for parathyroid gland development. Although all 3 have the same *GCM2* variant, they exhibit varying degrees of hypocalcemia and require different doses of treatment, reflecting the heterogeneity of this disease.

Teriparatide is the active fragment of PTH, which has a similar systemic half-life to full-length PTH. Teriparatide is approved for treatment in adults with osteoporosis but has not been Food and Drug Administration approved for use in adults or children with HP. Forteo (PTH 1-34) is widely used off label as a treatment of HP [10]. Further, teriparatide has been used to treat HP in neonates and infants [11, 12], including 1 infant with the *GCM2* pathogenic variant [2].

This case highlights the severity of hypocalcemia that can present in FIH as well as the difficulty of maintaining normocalcemia with conventional treatment, especially in the setting of illness. Teriparatide proved to be a quick and effective treatment in our case.

Another interesting lesson from this case is the evolving nature of our understanding of genetic variants. Initially, the *CASR* R990G variant was thought to be the cause of the patient's hypocalcemia because the siblings exhibited this variant and exhibited hypocalcemia and hypercalciuria despite the report stating that this sequence change is mildly activating and causal of hypercalciuria but not usually resulting in hypocalcemia. However, genetic testing for our patient returned as negative. In the 3 years between an initial *CASR* sequencing test in the eldest sibling and our patient, the *CASR* R990G variant had been reclassified as a benign variant per the American College of Medical Genetics and Genomics and the Association for Molecular Pathology due to its high allele frequency in the general population and additional evidence showing conflicting impact on renal Ca excretion [13]. Only upon contacting the genetic laboratory was the presence of the variant confirmed to also exist in our proband. Pursuing comprehensive genetic testing eventually revealed the accurate diagnosis for this family, and the uncertainty of the previously reported *CASR* variant caused delays in diagnosis and optimization of treatment. This serves as a valuable reminder that genetic results should occasionally be reassessed, particularly in cases where the clinical picture is not clear. It also emphasizes that identifying a variant in a gene associated with the patient's phenotypic presentation does not establish causality despite the statistical improbability of their co-occurrence.

In summary, we report a novel pathogenic variant in the *GCM2* gene providing further evidence of its role in Ca homeostasis. Additionally, we report that patients with isolated HP can be managed with teriparatide without any adverse outcomes to date.

## Learning Points

- Familial isolated HP is a rare and heterogenous disease that can be sporadic or inherited.
- The *GCM2* gene plays an important role in Ca homeostasis.
- Teriparatide is a safe and effective treatment in children with FIH.
- Unremarkable genetic studies should occasionally be reassessed, particularly in cases where the clinical picture is not clear.

## Contributors

A.T. and S.D.M. were involved in the diagnosis and management of the patient and the writing of the manuscript. J.A.M.-A. and J.K.S. provided genetics expertise in data interpretation. K.K.W. provided scientific insight and helped with patient management. All authors reviewed and approved the manuscript.

## Data Availability

Data sharing is not applicable to this article as no datasets were generated or analyzed during the current study.
